# Anesthetic Management for Pediatric Lung Resection: A Single-Center Retrospective Analysis

**DOI:** 10.7759/cureus.76138

**Published:** 2024-12-21

**Authors:** Pedro Gonçalves, Amélia Ferreira, Rita Aguiar, Rita Fernandes, Marta Guerra

**Affiliations:** 1 Anesthesiology, Centro Hospitalar Universitário de São João, Porto, PRT

**Keywords:** anesthetic management, lung resection, pediatric anesthesiology, single-center study, thoracotomy, video-assisted thoracoscopic surgery (vats)

## Abstract

Background

Lung resection is a complex surgical procedure performed in children to address various pulmonary conditions. The success of this surgical intervention in these patients lies in a multidisciplinary approach, with anesthetic management playing a critical role in ensuring the safety and efficacy of the procedure.

Methods

After approval by the local ethics committee, clinical data of 17 pediatric patients who underwent lung resection in our hospital from January 2012 to December 2022 were retrospectively analyzed.

Results

A total of 16 patients underwent video-assisted thoracoscopic surgery (VATS), and only one patient underwent open thoracotomy. Most indications for this type of procedure are linked to cystic disease at a stage where respiratory function is not severely compromised. Lung resection procedures were conducted under general anesthesia and invasive blood pressure monitoring in all patients. Various methods, such as double-lumen endotracheal tubes (DLTs), bronchial blockers (BBs), and specialized endobronchial devices, were employed to selectively collapse the lung undergoing resection while maintaining ventilation in the contralateral lung. A multimodal analgesia regimen, combining systemic analgesia with regional anesthesia techniques (epidural or regional trunk blocks), was employed.

Conclusions

Thorough preoperative preparation is critical for a desirable outcome in pediatric patients undergoing lung resection. The need for one-lung ventilation (OLV) presents challenges, especially in younger ages, where this surgery is more commonly performed. The use of BBs is often necessary, along with the likelihood of their extraluminal application. In recent years, to reduce the risks associated with thoracic epidural analgesia, regional trunk blocks have been described as a safe and effective alternative in pain management.

## Introduction

Lung resection in children is a complex surgical procedure to treat various conditions such as cystic pulmonary adenomatoid malformation, congenital lobar emphysema, chronic infections, and malignancies. Recent developments in minimally invasive surgical techniques have increased the use of video-assisted thoracoscopic surgery (VATS) in pediatric patients.

Anesthetic management plays a pivotal role in ensuring patient safety and optimizing surgical outcomes. A key component during pediatric lung resection is effective one-lung ventilation (OLV). The physiological challenges of OLV in children, including their reduced functional residual capacity and susceptibility to ventilation-perfusion mismatch, add complexity to the process. However, advancements in lung isolation techniques and improvements in device design have significantly enhanced the safety and feasibility of these procedures, even in small children [1, 2-4].

The increasing adoption of minimally invasive techniques, like VATS, highlights the growing demand for effective OLV strategies in pediatric thoracic surgery. There is also a growing recognition of the role of regional anesthetic techniques in postoperative pain management, beyond traditional local infiltration and neuraxial approaches [2-4].

This article provides a brief review of current airway management strategies for OLV and analgesia in children undergoing thoracic surgery, as well as our experience during perioperative and postoperative periods, based on a single-center retrospective analysis. It is also a comprehensive understanding of this critical area in pediatric anesthesiology.

## Materials and methods

Ethical approval

The study received approval from the ethics committee of the Centro Hospitalar Universitário de São João, Porto, Portugal (approval number CE-274-22). Access was granted to the surgical procedures database performed at the hospital center from January 2012 to December 2022. Due to the retrospective nature of this study, patient consent was not considered necessary.

Study design and group

**Figure 1:**
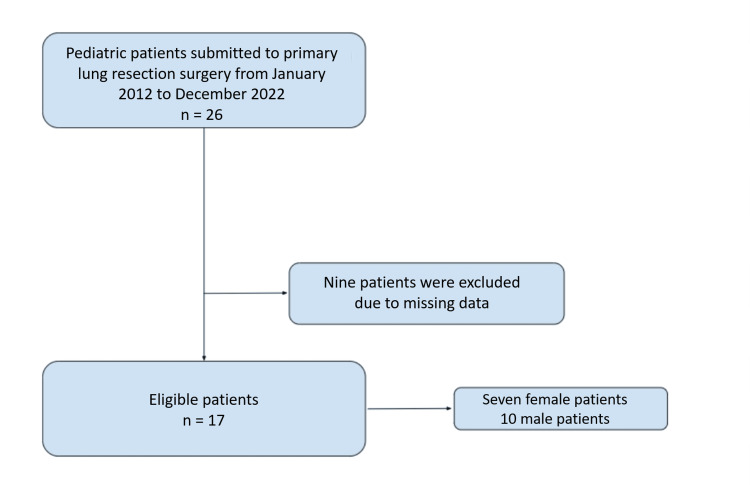
Flowchart of the exclusion criteria

This retrospective study included pediatric patients who underwent lung resection, preoperative evaluation, intraoperative anesthetic management, and postoperative outcomes were considered. Between January 2012 and December 2022, 26 patients were submitted to primary lung resection under anesthesia at our center. The sole exclusion criterion was the absence or incompleteness of data. Following the application of the inclusion and exclusion criteria, 17 patients were deemed eligible for analysis (Figure 1).

Patient data was extracted from electronic medical records. The following patient information was collected: age, American Society of Anesthesiology (ASA) status, gender, diagnosis, type of surgical procedure, details of preoperative assessment, type of anesthesia, lung isolation technique, intraoperative and postoperative analgesia regimen data, and information on complications. All postoperative complications were recorded, along with the number of days until hospital discharge.

Descriptive statistics

Demographic, clinical, and procedural data were summarized using appropriate descriptive statistics. All analyses were performed using R software, version 4.4.2 (R Foundation for Statistical Computing, Vienna, Austria). 

## Results

Patients’ characteristics

The majority of patients included were up to three years of age, consistent with the fact that most of the pulmonary pathology referred to our center is of congenital etiology. In most cases, children were asymptomatic or had a history of recurrent respiratory infections. In three of these cases, some degree of respiratory failure was observed, and they were classified as ASA grade III. However, no comorbidities other than respiratory concerns were observed (Table 1).

**Table 1:** Patient characteristics ASA: American Society of Anesthesiology

		N
Age (years)	0-1	5 (29%)
	1-3	9 (53%)
	3-5	1 (6%)
	>5	2 (12%)
ASA grade	I	2 (12%)
	II	12 (70%)
	III	3 (18%)
Gender	Female	7 (41%)
	Male	10 (59%)

Preoperative evaluation

A comprehensive preoperative assessment is crucial to evaluate the child's overall health, respiratory function, and any associated comorbidities. In cases of congenital lung abnormalities, like pulmonary sequestration or congenital cystic adenomatoid malformation, assessing any associated anomalies is considered to be essential. In this context, a routine examination, hematology, biochemistry, chest X-ray (CXR), and a thoracic CT scan were performed. Symptomatic patients or those with compromised respiratory function underwent additional pulmonary function tests.

The majority of cases (15) were referred due to congenital cystic disease. Pulmonary abscess and extralobar sequestration were responsible for the remaining two referred cases. Lobar and sublobar resections were the surgical procedures of choice in most of these cases (Table 2).

**Table 2:** Summary of diagnosis and surgical procedures VATS: video-assisted thoracoscopic surgery

		N
Diagnosis	Congenital cystic adenomatoid malformation	15 (78%)
	Lung abscess	1 (6%)
	Extralobar pulmonary sequestration	1 (6%)
Surgical procedure	Left lower lobectomy (VATS)	4 (24%)
	Left lower lobectomy (thoracotomy)	1 (6%)
	Right upper lobectomy (VATS)	1 (6%)
	Right middle lobectomy (VATS)	1 (6%)
	Right lower lobectomy (VATS)	8 (48%)
	Resection of pulmonary sequestration (VATS)	1 (6%)
	Right lower sublobar resection (VATS)	1 (6%)

Intraoperative evaluation

Lung resection procedures were conducted under general anesthesia for all patients, with a predominant utilization of VATS in 16 out of the 17 cases included in this study. Open thoracotomy was performed in one case. Intraoperative monitoring included electrocardiogram (ECG), noninvasive blood pressure (NIBP), pulse oximetry (SpO2), end-tidal CO2 (ETCO2), body temperature (TºC), neuromuscular blockade monitoring using a train-of-four (TOF), and urine output.

An inhalational induction was performed with sevoflurane, and after securing intravenous access, a bolus of fentanyl (2-3 µg/kg) and rocuronium (0.6 mg/kg) was administered to facilitate tracheal intubation. In cases where intravenous access was readily available or established, an intravenous induction with fentanyl and propofol was the preferred approach.

The maintenance of adequate anesthetic depth was conducted with the support of Bispectral Index (BIS) monitoring.

An arterial line for invasive blood pressure (IBP) monitoring was placed after anesthesia induction, contributing to comprehensive hemodynamic management during all the procedures.

In just one case, it became necessary to use a norepinephrine infusion, which was discontinued at the end of surgery. Notably, in four cases, a single bolus of ephedrine or phenylephrine was required to maintain hemodynamic stability. In the remaining 12 cases, no vasopressors were needed.

For cases presenting challenging airway anatomy, the availability of specialized equipment and expertise for airway rescue maneuvers played a pivotal role, including fibroscopy and videolaryngoscopy.

In our retrospective analysis, none of the 17 cases presented descriptions of a pre-existing difficult airway or clinical signs predicting such difficulties. The conventional laryngoscopy for endotracheal intubation was the primary approach used in all cases. In two of the cases, difficulties were reported in the first intubation attempt, both of which were successfully managed without desaturation or respiratory complications on the second attempt using conventional laryngoscopy.

The minimally invasive surgical techniques, particularly VATS, have gained popularity in pediatric patients. This has concurrently increased the demand for lung isolation techniques to enhance surgical exposure while preventing the contamination of the contralateral lung. Various methods, such as double-lumen endotracheal tubes (DLT), bronchial blockers (BB), and specialized endobronchial devices, have been employed to selectively collapse the lung undergoing resection while maintaining ventilation in the contralateral lung.

Analyzing our cases, 15 out of 17 patients were below four years old. For these younger patients, BBs were used in seven cases, a Fogarty embolectomy catheter was used in two cases, and selective intubation with a single-lumen endotracheal tube (ETT) was an option in five cases. (Table 3)

**Table 3:** Type of anesthesia and type of OLV OLV: one lung ventilation; ETT: endotracheal tube

		N
Type of anesthesia	General anesthesia	5 (29%)
	Combined general anesthesia with thoracic epidural	12 (71%)
Type of OLV	Single lumen ETT	5 (29%)
	Bronquial blocker	7 (41%)
	Double lumen tube	2 (12%)
	Fogarty embolectomy catheter	2 (12%)
	No need for OLV	1 (6%)

In two cases, OLV was carried out using a Fogarty embolectomy catheter as a surrogate for BB before BB was available at our institution. Currently, the practice has shifted towards the use of Fuji Uniblocker (Ambu Inc., Columbia, MD, USA), and all seven remaining cases where a BB was used were performed using this device.

Extraluminal placement of the BB was necessary in five cases, all involving children under two years old, in which small ETTs (<5 mm) were employed. In a notable case, a thoracotomy in a newborn at 21 days of life was performed without the need for OLV. Anesthesia for this procedure was conducted using a single-lumen ETT.

In the two cases involving patients above the age of seven, a DLT was employed to facilitate OLV. The choice of OLV techniques was tailored to the patient's age, anatomical considerations, the specific requirements of the surgical procedure, and the availability of devices at the moment in our center. 

The correct positioning of the ETT, Fogarty embolectomy catheter, BB, and DLT was ensured through fiberoptic bronchoscopy.

In light of the extensive nature of pediatric lung resection and the potential for significant intraoperative nociception, effective pain management strategies were employed. In the initial 12 cases of our sample, a combination of general anesthesia and low (T7-T11) thoracic epidural analgesia was adopted. A single bolus of 0.03-0.05 ml per dermatome per kg of 0.2% ropivacaine or 0.25% levobupivacaine was administered at the beginning of the procedure, followed by a continuous infusion of local anesthetics (0.1%-0.2% ropivacaine or 0.0625%-0.125% levobupivacaine) at 0.2-0.4 mg/kg/h.

A systemic analgesia regimen was applied to all patients, containing paracetamol (15 mg/kg) and ketorolac (0.5 mg/kg). In cases where epidural analgesia was not used, SOS morphine boluses (0.1 mg/kg) were added.

Postoperative outcomes and analgesia strategies

Post surgery, 16 out of the 17 patients were safely extubated after the reversal of neuromuscular blockade and transferred to the pediatric intensive care unit. One patient remained under mechanical ventilation due to an ongoing respiratory infection associated with congenital cystic adenomatoid malformation, leading to an extended hospitalization in our pediatric intensive care unit and pediatric ward.

The most frequent surgical complication in the postoperative period was mild subcutaneous emphysema, observed in two cases, both successfully treated conservatively.

Within our patient sample, three cases experienced accidental exteriorization of the epidural catheter on the first day postoperatively; in these cases, systemic analgesia was adjusted, and no adverse events were observed. In the remaining patients, the thoracic epidural catheter remained in situ for three to five days, with continuous infusion of local anesthetic, combined with paracetamol and ketorolac for analgesia.

## Discussion

Pediatric lung resection presents distinctive challenges in anesthesia management due to the intricacies of pediatric physiology and anatomy and the complexity of the surgery itself. The success of this procedure relies not only on surgical expertise but also on following a comprehensive and tailored anesthetic approach [1].

Before initiating anesthesia, a thorough preoperative assessment is paramount. Pediatric patients need meticulous evaluation of their medical history, airway, respiratory function, and cardiovascular status. Comprehensive monitoring of vital signs, including invasive arterial blood pressure, is essential during lung resection surgery. Establishing adequate intravenous access is a prerequisite, and fluid management should be carefully tailored to maintain optimal intravascular volume, preventing excessive fluid administration that could exacerbate lung edema and compromise oxygenation. One-lung ventilation can influence hemodynamics through changes in pulmonary blood flow and altered intrathoracic pressures. Therefore, close monitoring and effective management of hemodynamic parameters are crucial to prevent hypotension and cardiovascular compromise.

One-lung ventilation involves selective ventilation of one lung while allowing collapse or deflation of the other, facilitating surgical access and manipulation within the thoracic cavity. Commonly employed in various thoracic surgeries, including lung resections, esophagectomies, and thoracoscopic procedures, OLV enhances surgical visualization and minimizes the risk of contamination from the non-operative lung [2]. The straightforward method for lung isolation is to place an ETT into the desired bronchus. Isolation of the right mainstem bronchus is easier than the left due to the anatomical differences between them. However, this simple isolation technique doesn’t allow suction or deliver continuous positive airway pressure to the desired lung. Adequate collapse of the excluded lung is therefore more challenging [2-5].

Double-lumen endotracheal tubes remain the gold standard for achieving OLV. These specialized tubes have two lumens, separately entering the right or left bronchus, to facilitate selective lung isolation. Proper positioning is crucial and is confirmed via fiberoptic bronchoscopy to ensure adequate placement and prevent complications like contralateral lung collapse or airway trauma [2-5].

Bronchial blockers offer an alternative to DLTs, especially in scenarios where DLT insertion may be challenging or contraindicated. These devices, inserted through a single-lumen ETT, occlude the mainstem bronchus or bronchial orifices selectively, allowing OLV. The placement of BBs requires precise positioning under fiberoptic guidance for effective lung isolation [2-5]. Despite the increased use of VATS, the experience of OLV in very young children is scarce and remains a challenge to the anesthesiologist. Techniques for OLV in pediatric patients include several devices, each of them with its own limitations, and one must adapt to the possibilities routinely available in the institution. More often, a DLT tube is used in children >8 years, and children under eight years of age generally require the use of a BB, and a small-sized ETT usually requires extraluminal placement of the BB. The intraluminal placement of a BB allows for the better fixing of the BB and diminishes the morbidity associated with displacements during surgery. Malpositioning of BB can lead to contralateral lung collapse, inadequate isolation, or airway trauma [4-5].

**Figure 2:**
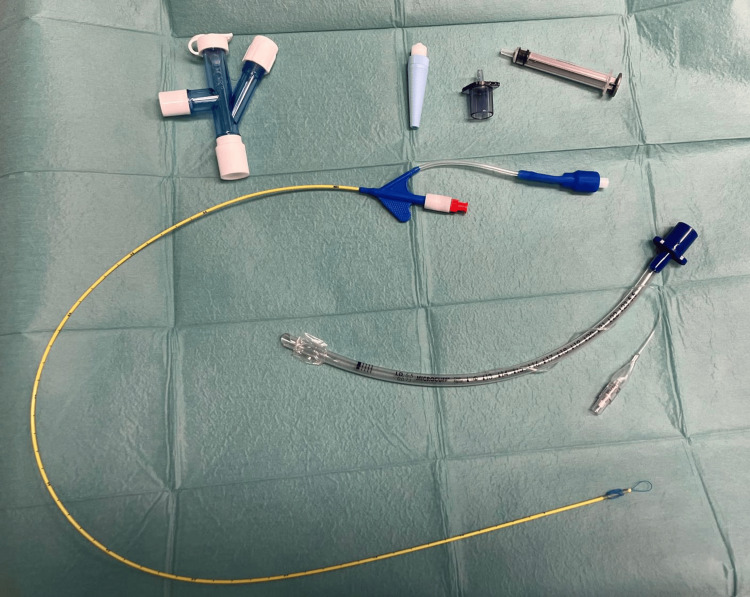
Arndt endobronchial blocker

**Figure 3:**
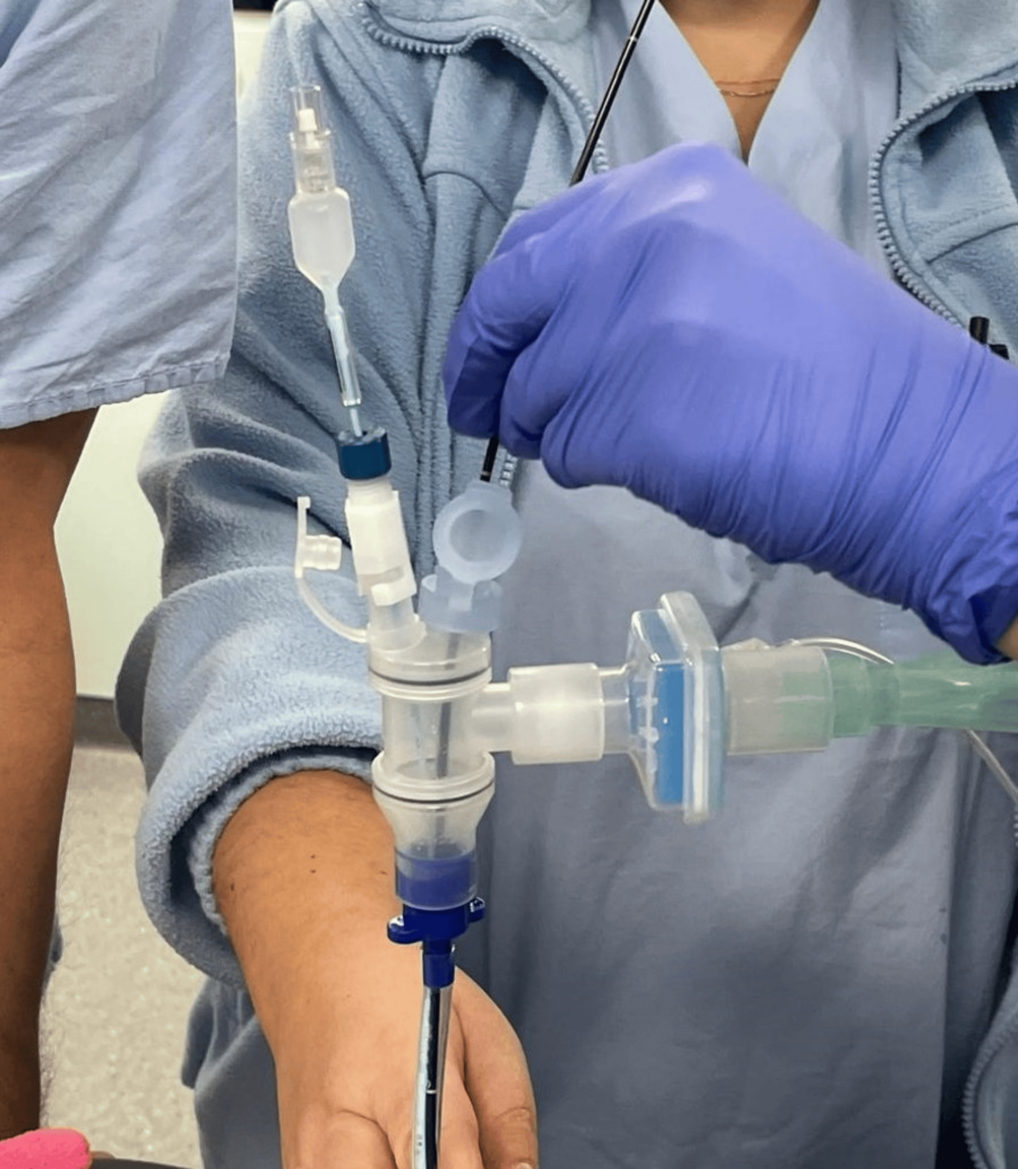
An intraluminal Uniblocker in a 5.0 endotracheal tube

The Univent BB incorporates a separate lumen allowing for independent lung ventilation. Its design includes an endobronchial cuff that can be inflated to seal the bronchus, ensuring lung isolation. The separate lumen permits the ventilation of the unblocked lung. The Arndt endobronchial blocker employs a balloon-tipped catheter design and enables selective lung isolation by inflating the balloon within the desired bronchus. It offers flexibility in navigating bronchial anatomy and can be used with fiberoptic guidance for precise positioning (Figure 2). Fuji Uniblocker uses a balloon occluder attached to a flexible catheter and achieves lung isolation by inflating the balloon within the bronchus, blocking airflow to the designated lung while maintaining ventilation to the contralateral lung (Figure 3) [5-6]. A Fogarty embolectomy or Foley catheter has been used as a BB when children require one-lung ventilation. The catheter can be passed either through or alongside the endotracheal tube; however, one of the main disadvantages (especially when extraluminal placement is necessary) is that the blocker may be easily dislodged. The catheter is usually inserted into an endotracheal tube through a right-angle connector with a self-sealing diaphragm or suction port [7].

Effective pain management during and after VATS is crucial for patient comfort, facilitating early ambulation, and minimizing postoperative complications. The minimally invasive nature of VATS, characterized by small incisions and the use of a thoracoscope, results in reduced tissue trauma and postoperative pain compared to thoracotomy. Patients undergoing VATS generally require less aggressive analgesia owing to the minimized surgical insult. The use of epidural analgesia in this population requires a delicate balance between providing efficient pain relief and mitigating potential complications. Complications associated with epidural analgesia extend beyond technical challenges. Epidural analgesia offers excellent pain relief with reduced opioid-related side effects such as nausea, sedation, and respiratory depression. However, its use poses inherent risks and challenges, especially in pediatric patients. Epidural catheter placement carries a risk of inadvertent vascular puncture, leading to hematoma formation or bleeding, particularly in children with a smaller anatomy and less tissue coverage. External forces or movement can cause catheter dislodgement or breakage, interrupting medication delivery or leading to catheter fragments remaining within the epidural space, thereby increasing the risk of complications. Accidental exteriorization is also more frequent in children, as seen in our sample. Catheter migration in active children can further complicate its position, impacting analgesic efficacy. Prompt recognition of issues like infection, dislodgment, or migration allows for immediate intervention, potentially averting severe consequences [8].

Intercostal nerve blocks involve the administration of local anesthetics directly into the intercostal spaces, providing targeted analgesia to the surgical site. Surgeons often perform these blocks after VATS to alleviate immediate postoperative pain. The strategy of multimodal analgesia plus intercostal nerve blocks also poses a lower risk of hypotension, motor blockade, or nerve damage compared to epidural analgesia. The risk-benefit profile associated with satisfactory pain control in the postoperative period has led to a change in our practice [9].

The evidence in the latest medical literature regarding analgesic techniques in VATS for adults highly recommends the use of multimodal analgesia supplemented with regional analgesia techniques such as the paravertebral block, erector spinae plane block (ESPB), or the serratus anterior plane block [10]. An ESPB involves the deposition of local anesthetic in the plane between the erector spinae muscle and the transverse processes of the vertebrae. This technique provides analgesia by blocking the dorsal and ventral rami of the spinal nerves, offering coverage to a broader dermatomal distribution. An ESPB provides effective analgesia for thoracic surgeries, including lung resection, by targeting the dorsal rami of the thoracic nerves. Its main advantage lies in the ease of performance, safety, and potential for reduced opioid consumption postoperatively. To achieve maximal satisfaction and postoperative pain control, we started to perform this block in a few recent cases. However, findings indicate that an ESPB might offer less segmental specificity, in addition to the spread of local anesthetic in an ESPB being somewhat unpredictable. Also, despite its growing popularity, the evidence base supporting ESPB is limited compared to more established techniques [11-12].

Postoperative care of pediatric patients undergoing lung resection involves a smooth transition from the effects of anesthesia to wakefulness, ensuring the child's comfort and stability. Extubation timing is crucial, balancing the need to maintain airway patency and respiratory function while avoiding unnecessary prolonged ventilation. In our experience, in the majority of these situations, it is possible to perform safe and early extubation.

Based on these paradigms, the current anesthetic approach in our center combines general anesthesia with the placement of a perineural catheter in the erector spinae plane along with multimodal systemic analgesia (Table 4).

**Table 4:** Current anesthetic approach ASA: American Society of Anesthesiology; ESPB: erector spinae plane block; IV: intravenous; ICU: intensive care unit; OLV: one-lung ventilation

Anesthesia	General anesthesia combined with an ESPB (T7-T8)
Monitoring	ASA standard monitoring plus monitoring of the anesthesia depth, neuromuscular blockade, and invasive arterial blood pressure for hemodynamic assessment
OLV strategy	For younger patients: bronchial blocker
	For patients above 7/8 years old: double-lumen tube
Analgesia strategy	IV acetaminophen 15 mg/kg 6/6h
	IV ketorolac 0.5 mg/kg
	IV morphine 0.1 mg/kg
	0.3-0.5 ml/kg 0.2% ropivacaine in the perineural catheter
Postoperative strategy	Extubation and transfer to the ICU ward
	Multimodal opioid spare analgesia consisting of IV acetaminophen 15 mg/kg 6/6h plus
	IV ketorolac 0.5 mg/kg 8/8h plus
	Continuous infusion 0.1-0.2 ml/kg/h 0.1-0.2% ropivacaine in perineural catheter plus
	SOS boluses of 0.2 to 0.3 ml/kg 0.2% ropivacaine in perineural catheter

This retrospective analysis provides valuable insights into the anesthetic management of pediatric lung resection. However, several limitations must be acknowledged. A single-institution study may cause a bias, as practices, equipment availability, and protocols may vary across centers. Additionally, the sample size is relatively small due to the rarity of the procedure, and the population is highly heterogeneous. Also, the study's retrospective design depends on the accuracy and completeness of existing medical records. One-lung ventilation presents unique challenges in managing hypoxemia, especially in the pediatric population. The fact that the analysis is retrospective and based on electronic records hinders the accurate assessment of these intraoperative complications. Future research should focus on prospective, multicenter studies with larger sample sizes to validate the findings and assess long-term outcomes, enhancing our understanding and improving care for young children undergoing thoracic surgery.

## Conclusions

Pediatric lung resection poses unique challenges for the anesthesiologist. Although most indications for this type of procedure are linked to cystic disease at a stage where respiratory function is not severely compromised, the possibility of the existence of previous respiratory disease should be considered, thereby making preoperative assessment essential. The need for OLV for the surgical approach presents challenges regarding airway management, especially in younger ages, where this surgery is more commonly performed. The use of BB is often necessary, along with the likelihood of their extraluminal application. It should be noted that practices related to postoperative analgesia techniques have been evolving in recent years to reduce the risks associated with epidural analgesia while taking advantage of the effectiveness and increased safety of regional trunk blocks.
